# Developing a RadLex-Based Named Entity Recognition Tool for Mining Textual Radiology Reports: Development and Performance Evaluation Study

**DOI:** 10.2196/25378

**Published:** 2021-10-29

**Authors:** Shintaro Tsuji, Andrew Wen, Naoki Takahashi, Hongjian Zhang, Katsuhiko Ogasawara, Gouqian Jiang

**Affiliations:** 1 Department of Health Sciences Research Department of Radiology Rochester, MN United States; 2 Graduate School of Health Sciences Hokkaido University Sapporo Japan; 3 Department of Radiology Mayo Clinic Rochester, MN United States

**Keywords:** named entity recognition (NER), natural language processing (NLP), RadLex, ontology, stem term

## Abstract

**Background:**

Named entity recognition (NER) plays an important role in extracting the features of descriptions such as the name and location of a disease for mining free-text radiology reports. However, the performance of existing NER tools is limited because the number of entities that can be extracted depends on the dictionary lookup. In particular, the recognition of compound terms is very complicated because of the variety of patterns.

**Objective:**

The aim of this study is to develop and evaluate an NER tool concerned with compound terms using RadLex for mining free-text radiology reports.

**Methods:**

We leveraged the clinical Text Analysis and Knowledge Extraction System (cTAKES) to develop customized pipelines using both RadLex and SentiWordNet (a general purpose dictionary). We manually annotated 400 radiology reports for compound terms in noun phrases and used them as the gold standard for performance evaluation (precision, recall, and F-measure). In addition, we created a compound terms–enhanced dictionary (CtED) by analyzing false negatives and false positives and applied it to another 100 radiology reports for validation. We also evaluated the stem terms of compound terms by defining two measures: occurrence ratio (OR) and matching ratio (MR).

**Results:**

The F-measure of cTAKES+RadLex+general purpose dictionary was 30.9% (precision 73.3% and recall 19.6%) and that of the combined CtED was 63.1% (precision 82.8% and recall 51%). The OR indicated that the stem terms of *effusion*, *node*, *tube*, and *disease* were used frequently, but it still lacks capturing compound terms. The MR showed that 71.85% (9411/13,098) of the stem terms matched with that of the ontologies, and RadLex improved approximately 22% of the MR from the cTAKES default dictionary. The OR and MR revealed that the characteristics of stem terms would have the potential to help generate synonymous phrases using the ontologies.

**Conclusions:**

We developed a RadLex-based customized pipeline for parsing radiology reports and demonstrated that CtED and stem term analysis has the potential to improve dictionary-based NER performance with regard to expanding vocabularies.

## Introduction

### Background

The widespread adoption of electronic medical record (EMR) systems in recent years has increasingly brought opportunities to research communities regarding the secondary use of EMR data such as medical images and clinical notes [1] to support clinical and translational research. It is expected that real-world data will contribute to generating medical evidence, optimizing the use of medical resources, and creating high-quality diagnostic or treatment guidelines [2,3]. To establish effective retrieval and extraction of such data stored in the EMR, standard codes are usually used to describe patient records and make them computable and interpretable. For example, the *International Classification of Diseases* is a standard code system used to classify diseases or diagnoses for medical records [4]. The *International Classification of Diseases* can be used to identify classified disease names from medical records in information-retrieval applications. In addition, a standard code system can also be used to extract features from medical texts such as pathology reports, radiology reports, and family history reports. For example, SNOMED-CT (Systematized Nomenclature of Medicine-Clinical Terms) is a standard terminology in the field of medical care [5], which is often used as a resource for the automatic named entity recognition (NER) of medical texts [6]. Moreover, SNOMED-CT is formalized as an ontology, which has a hierarchical structure of terms and semantic relationships between terms. Such an ontology supports medical reasoning with standard concept definitions and axioms among concepts.

In the field of radiology, a large amount of medical imaging data and diagnostic reporting data is stored in the EMR, which has become an important data source for acquiring knowledge. The use of standard code systems is critical for the effective mining of the data source. RadLex, produced by the *Radiological Society of North America*, is a controlled-standard biomedical ontology that provides codes, conceptual relationships, and procedures of imaging examinations [7]. RadLex was historically developed as indexing teaching files for radiologists, provided by the *American College of Radiology* [8]. Currently, RadLex is widely used to support the creation of templates for generating radiology reports [9], mining radiology reporting data [10], indexing medical images and reports [11], and standardizing examination descriptions [12]. From the perspective of data interoperability in the radiology domain, RadLex is a unique ontology in that it enables semantic parsing of free-text radiology reports by playing a role in integrating identified entities into a higher-level semantic concept such as *anatomical entities*, *clinical findings*, *imaging observation*, and *procedures*.

NER is usually used for preprocessing unstructured data for machine learning research, for example, extracting features from radiology reports [13]. In a previous study on the NER evaluation based on radiologist agreement, it was reported that the F-measure of dictionary-based NER was lower than that of conditional random fields (CRFs) [14,15] and rule-based natural language processing (NLP) [16,17]. However, machine learning–based NER does not provide a relationship between terms, and the reason for the F-measure of dictionary-based NER being lower than that of machine learning–based NER is that it is difficult to identify various patterns of compound terms using standard terminologies or ontologies. For example, in the case of the compound term *right-sided IJ central venous catheter*, all the words in the term except for *catheter* are modifiers. In short, there are several patterns such as *IJ* *central venous catheter* and *venous catheter* that can be identified as annotations by radiologists.

### Objective

Although an ontology such as RadLex can be leveraged to enhance data interoperability and track relationships and hierarchical structure, we consider that the ontology should also be applied to improve the NER of compound terms in radiology reports. However, few studies have been conducted to evaluate the coverage of RadLex for the NER of compound terms for mining radiology reports. To evaluate and extend the coverage of the lexicon for extracting features from radiology reports, the aim of this study is to develop and assess an NER tool based on RadLex, explore the entities included in RadLex, and subsequently extend the ontology for a higher F-measure on feature extraction by dictionary-based NER.

## Methods

### RadLex Features

RadLex is a controlled-standard biomedical ontology produced by the *Radiological Society of North America*, which provides unique codes, conceptual mapping based on hierarchal structure, and procedures of imaging examinations [7]. We used and analyzed RadLex version 1.3.4 [18], which includes 46,434 primary terms and 42,831 compound terms.

### General Purpose Dictionary

We used a general purpose dictionary (GPD), SentiWordNet [19], to compare RadLex coverage with a general dictionary. SentiWordNet, which is a GPD for sentiment analysis in the context of social network services, provides a negative or positive score of terms. The number of words of parts of speech (POS) is 117,659, including 82,115 distinct nouns, 13,767 verbs, 18,156 adjectives, and 3621 adverbs. The number of compound terms is 48,469.

### Clinical Text Analysis Knowledge Extraction System

The clinical Text Analysis Knowledge Extraction System (cTAKES), which is an NLP system for extraction of information from EMR clinical free text, contains an automatic NER tool using a dictionary lookup mechanism [20]. The default dictionary of cTAKES is based on the Unified Medical Language System (UMLS) [21] and provides annotations of diseases or disorders, signs or symptoms, anatomical sites, procedures, and medications. For example, the dictionaries based on SNOMED-CT and RxNORM, which is part of the UMLS, cover the fields of general clinical findings and medications. We investigated the compound terms in each dictionary for the analysis.

### Medical Information Mart for Intensive Care-III

The Medical Information Mart for Intensive Care-III (MIMIC-III) is a free, open database provided by the Massachusetts Institute of Technology Laboratory for Computational Physiology, which includes approximately 60,000 deidentified admissions of patients at the Beth Israel Deaconess Medical Center from 2001 to 2012 [22]. Using PostgreSQL, we queried the note events table of the MIMIC-III database, which includes approximately 520,000 radiology reports.

### Procedures

**Figure 1:**
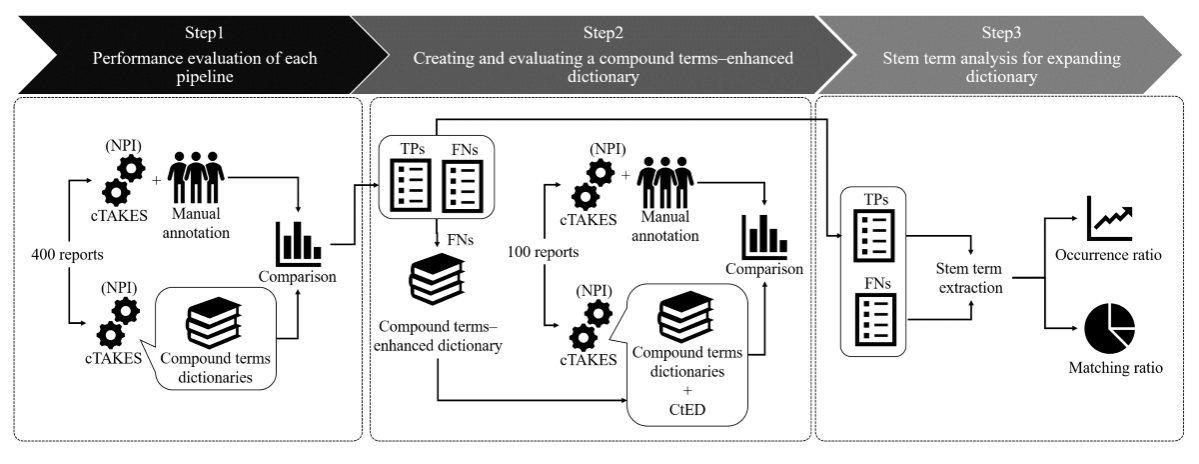
Overview of methods. cTAKES: clinical Text Analysis and Knowledge Extraction System; CtED: compound terms–enhanced dictionary; FN: false negative; NPI: noun phrase identification; TP: true positive.

The overall goal of our study is to clarify the coverage of RadLex-based dictionaries with compound terms and to construct and evaluate the NER tools that use the RadLex-based dictionaries for mining free-text radiology reports. First, we customized cTAKES to build the RadLex and GPD dictionaries. As previously mentioned, the default dictionaries of cTAKES provided by the UMLS are SNOMED-CT and RxNORM. Second, we combined these three dictionaries in the following patterns: Default, Default+RadLex, and Default+RadLex+GPD. Third, we removed single terms from each dictionary and evaluated their performance. Finally, we carried out the three processes of analysis (step 1 to step 3) to obtain profiles of the stem terms for improving the performance of NER (Figure 1).

### Creating Annotation Corpus of Radiology Reports

We randomly selected 400 reports of computed tomography (CT), magnetic resonance imaging (MRI), positron emission computed tomography (PET), and radiography (x-ray) from the MIMIC-III database (100 reports for each imaging modality type). These reports were in a free-text format and were categorized into sections; we used the *Findings*, *Interpretations*, and *Impressions* sections, which play a core role in diagnosis. There were 28.9 sentences per report and 179.1 tokens per report. An additional 100 reports (25 reports for each imaging modality type) were randomly selected and used in the validation study for compound terms.

We first conducted stop word removal and exchanged all the characters to the lower case. Next, we leveraged the AggregatePlaintextProcessor of cTAKES to identify noun phrases in the radiology reports so that we could perform a manual annotation for noun phrases. Next, we applied manual reviews to annotate compound terms. The compound terms were also tagged with all conceivable patterns based on the *stem term*. For example, the compound term *right upper lobe* is divided into *right upper lobe* and *upper lobe*. After the annotation, we can obtain two compound terms from *right upper lobe*. In this case, we defined *lobe* as a stem term. We also separated *right upper lobe of lung base* into *right upper lobe* and *lung base.* Thus, we defined the stem term as the modified term of a compound term in this study. These manual annotations were conducted and agreed on by 3 researchers with a background in radiology (n=1) and computer sciences (n=2). Generally, the annotations for compound terms are performed by expert radiologists and are agreed upon through discussion. As some studies have revealed that the annotation patterns of compound terms are different with institutions, we used all the patterns of compound terms as the gold standard.

### Developing a RadLex-Based NER Tool

First, we created a customized NER tool using cTAKES, which uses a dictionary lookup–based parser for NER. It extracts terms that can be looked up in the installed dictionary. Some previous studies have attempted to create customized dictionaries (eg, UMLS) [11] for NER, but few studies have investigated NER using RadLex for mining radiology reports [23]. In this study, we built a customized dictionary using RadLex as a domain-specific dictionary and SentiWordNet as a GPD. RadLex can be used to automatically extract technical terms from radiology reports [24], whereas SentiWordNet is usually used for sentiment analysis, which clarifies positive or negative descriptive text on social networking services. Moreover, we created dictionaries for compound terms. The terms of each dictionary were stored in the bar-separated value (BSV) file and located in the dictionary lookup–first directory, which allows the term to be extracted preferentially. cTAKES uses a SNOMED-CT and RxNORM dictionary by default. Finally, we created a collection of customized dictionaries in the following patterns: Default (SNOMED-CT and RxNORM), Default+RadLex, and Default+RadLex+GPD.

### Step 1: Performance Evaluation of Each Pipeline

For each customized pipeline, we evaluated the performance of four different sets of the three dictionary patterns using standard measures (ie, precision, recall, and F-measure). The formulas for the measures are as follows:


Precision = True positives / (True positives + False positives) × 100      **(1)**

Recall = True positives / (True positives + False negatives) × 100      **(2)**

F-measure = 2 × Precision × Recall / (Precision + Recall)      **(3)**

Here, true positive (TP) is defined as the number of manual annotations matched with the dictionary phrases, false positive (FP) is defined as the number of dictionary phrases matched with entities other than manual annotations, and false negative (FN) is defined as the number of annotations not matched with the dictionary phrases. We also evaluated the performance of four major imaging modalities: CT, MRI, x-ray, and PET. GATE (General Architecture for Text Engineering) developer version 8.4.1 [25] was used to compute these measures.

### Step 2: Creating and Evaluating a Compound Terms–Enhanced Dictionary

We also created a compound terms–enhanced dictionary (CtED) to improve performance (Figure 1). We added these compound terms to the FN category (as identified in the initial evaluation) in the custom dictionaries that were used for parsing 400 radiology reports. At the same time, we removed these compound terms in the FP category from these dictionaries. To validate the performance of the CtED, we carried out NER for another 100 radiology reports (25 reports for each imaging modality type; Figure 1). Finally, we calculated the precision, recall, and F-measure for the performance evaluation.

### Step 3: Stem Term Analysis for Expanding Dictionary

To obtain the full benefit of using RadLex, which is an ontology-based tool, we created 2 measures for a stem term. We first defined a measure called the occurrence ratio (OR) to determine the frequency of stem terms in TPs and FNs from step 2. The OR gives priority measures to add compound terms with stem terms into RadLex. For example, if the value of the OR for a stem term in TPs is high, it means that the number of compound terms (containing the stem term) that are correctly identified by the pipeline is high. In contrast, if the value of the OR for the stem term in FNs is high, it means that the number of compound terms (containing the stem term) that are identified as negative by the pipeline is high. Moreover, if a high OR stem with both TP and FN is identified, we can hypothesize that this stem shows that there is a high demand to extract the entity of reports but still lacks the compound terms having the stem. In short, the OR can visualize a profile of the demand and supply of stem term–oriented compound terms in the corpus.

Occurrence ratio (%) = Occurrence of a stem term in TP or FN / Total number of stem terms in TPs or FNs × 100%      **(4)**

Second, we defined a measure called the matching ratio (MR) to describe the distribution of stem terms in FNs that are matched with the dictionaries. The MR (%) was calculated using the formula presented below. The MR can guide the basic concept of the RadLex or SNOMED-CT (cTAKES default dictionary) concept that matches the stem terms. For example, if a stem term of *effusion* is found in RadLex, we continue to trace the parent concept until the concept is under the top hierarchy. Finally, we identified the concept of *clinical findings*. The MR provides the criteria for identifying the number of concepts. We used 15 concepts under the RadLex entity (ie, *anatomical entity*, *clinical finding*, *imaging modality*, *imaging observation*, *nonanatomical substance*, *object*, *procedure*, *process step*, *process*, *property*, *RadLex descriptor*, *RadLex nonanatomical set*, *report*, *report content*, and *temporary entity*). Each stem term was tracked using their upper-class ID (RadLex ID). For the cTAKES default dictionary, we used 19 concepts under the top class of SNOMED-CT (RxNORM was excluded because it does not have a hierarchal structure). The class are *Body structure*, *Clinical finding*, *Environment or geographical location*, *Event*, *Observable entity*, *Organism*, *Pharmaceutical/biologic product*, *Physical force*, *Physical object*, *Procedure*, *Qualifier value*, *Record artifact*, *Situation with explicit context*, *SNOMED-CT Model Component*, *Social context*, *Special concept*, *Specimen*, *Staging and scales*, and *Substance*. We manually checked all stem terms based on the criteria of the exact match through the BioPortal site (National Center for Biomedical Ontology) [26].

Matching ratio (%) = Occurrence of a stem term in FN matched with RadLex or SNOMED-CT / Total number of stem terms in FNs × 100%      **(5)**

## Results

### Performance Evaluation of Each Pipeline

The F-measure of the pipeline with the dictionaries Default+RadLex+GPD for compound terms was nearly the same as that of the pipeline with the dictionaries Default+RadLex (31.5% vs 31.4%; Table 1). In step 2—building and evaluating the CtED—the F-measures of the pipeline with the dictionaries Default+RadLex+GPD with and without the CtED were 63.1% and 30.9%, respectively (Table 2).

**Table 1:** F-measure, precision, and recall of each dictionary (step 1: number of reports=400).

Dictionaries	F-measure, %	Precision, %	Recall, %
Default	27.9	93.4	16.4
Default+RadLex	31.4	94.9	18.8
Default+RadLex+GPD^a^	31.5	93.2	19

^a^GPD: general purpose dictionary.

**Table 2:** F-measure, precision, and recall of each dictionary (step 2: number of reports=100).

Dictionaries	F-measure, %	Precision, %	Recall, %
Default+RadLex+GPD^a^ without enhancement	30.9	73.3	19.6
Default+RadLex+GPD with enhancement	63.1	82.8	51

^a^GPD: general purpose dictionary.

Regarding each imaging modality (Table 3), the F-measure of cTAKES+RadLex+GPD for x-ray was higher (64.3%) than that without enhancement (26.7%). The most frequent stem terms in the FNs were *effusion* (9.1% x-ray), *change* (3.5% CT), *change* (4.1% MRI), and *uptake* (12% PET; Table 4). The number of words in the compound terms in the FPs was mainly 2 (31,774/42,871, 74.12%), 3 (7876/42,871, 18.37%), and 4 (2271/42,871, 5.29%), which is approximately 97.78% (41,921/42,871) of all FNs.

**Table 3:** F-measure of the compound terms–enhanced dictionary of each modality.

Modality	cTAKES^a^+RadLex+GPD^b^ (%)	cTAKES+RadLex+GPD+CtED^c^ (%)
Computed tomography	33.5	62.4
MRI^d^	30.7	63.6
PET^e^	30.3	63.4
x-ray	26.7	64.3
All	30.9	63.1

^a^cTAKES: clinical Text Analysis and Knowledge Extraction System.

^b^GPD: general purpose dictionary.

^c^CtED: compound terms–enhanced dictionary.

^d^MRI: magnetic resonance imaging.

^e^PET: positron emission computed tomography.

**Table 4:** Top five occurrence ratios in each imaging modality.

Modality			Stem		OR^a^, n (%)
**Computed tomography**					
	**TP^b^ (n=1127)**				
		lobe		100 (8.87)	
		effusion		59 (5.24)	
		node		50 (4.44)	
		artery		39 (3.46)	
		hemorrhage		37 (3.28)	
	**FN^c^(n=3532)**				
		change		125 (3.54)	
		collection		98 (2.77)	
		lesion		95 (2.69)	
		effusion		94 (2.66)	
		evidence		69 (1.95)	
**MRI^d^**					
	**TP (n=840)**				
		artery		146 (17.38)	
		lobe		49 (5.83)	
		sinus		29 (3.45)	
		matter		20 (2.38)	
		body		20 (2.38)	
	**FN (n=3732)**				
		change		176 (4.72)	
		lesion		144 (3.86)	
		enhancement		132 (3.54)	
		evidence		95 (2.55)	
		study		89 (2.38)	
**PET^e^**					
	**TP (n=1123)**				
		node		192 (17.1)	
		lobe		102 (9.08)	
		gland		69 (6.14)	
		nodule		39 (3.47)	
		disease		36 (3.21)	
	**FN (n=4708)**				
		uptake		567 (12.04)	
		node		250 (5.31)	
		lesion		180 (3.82)	
		avidity		169 (3.59)	
		disease		157 (3.33)	
**x-ray**					
	**TP (n=323)**				
		effusion		46 (14.24)	
		tube		37 (11.45)	
		lobe		27 (8.36)	
		edema		18 (5.57)	
		lung		17 (5.26)	
	**FN (n=1279)**				
		effusion		117 (9.15)	
		tube		69 (5.39)	
		opacity		67 (5.24)	
		pneumothorax		62 (4.85)	
		line		57 (4.46)	

^a^OR: occurrence ratio.

^b^TP: true positive.

^c^FN: false negative.

^d^MRI: magnetic resonance imaging.

^e^PET: positron emission computed tomography.

In addition, the most frequent FPs that were removed from the cTAKES+RadLex+GPD dictionaries were *related to* (34/239, 14.2%), *abdomen and pelvis* (23/239, 9.6%), and *head and neck* (21/239, 8.8%).

### Most Frequent Stem Terms

**Figure 2:**
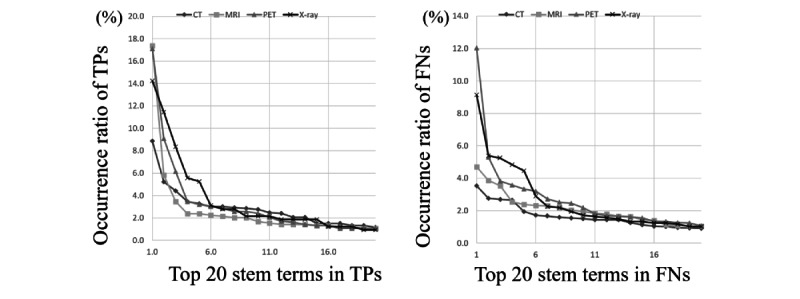
Occurrence ratio of true positives and false negatives in each imaging modality. CT: computed tomography; FN: false negative; MRI: magnetic resonance imaging; PET: positron emission computed tomography; TP: true positive.

The ORs of the TPs and FNs in each imaging modality (step 3) are shown in Figure 2. The stem terms of the TPs in the CT reports were more diverse than those in the MRI, PET, and x-ray reports. The FNs in the CT and MRI reports also showed the same trends. The most frequent stem terms in the TPs were *lobe* (100/1127, 8.87% CT), *artery* (146/840, 17.4% MRI), *node* (192/1123, 17.1% PET), and *effusion* (46/323, 14.2% x-ray; Table 4). In contrast, the most frequent stem terms in the FNs were *change* (125/3532, 3.54% CT), *change* (176/3732, 4.72% MRI), *uptake* (567/4708, 12.04% PET), and *effusion* (117/1279, 9.15% x-ray). Table 4 shows that stem terms such as *effusion*, *node*, *tube*, and *disease* had a need in both TPs and FNs.

Table 5 illustrates the distribution of the stem terms in FNs that are matched with a RadLex upper concept using the MR. The result of the MR was 71%, which included a connectivity of 47.4% with RadLex and 51.5% with the cTAKES default dictionary (SNOMED-CT and RxNORM). The stem terms that did not match RadLex and the cTAKES default dictionary accounted for 28.15% (3687/13,098). The matched classes in RadLex included clinical finding (1839/13,098, 14.04%), imaging observation (1508/13,098, 11.51%), and process (1000/13,098, 7.63%), and those in the cTAKES default dictionary included Body structure (1428/13,098, 10.9%), Over two category (1265/13,098, 9.66%), and Qualifier value (935/13,098, 7.14%).

**Table 5:** Classification of stem terms in false negatives based on cTAKES^a^, RadLex, and combined dictionary (n=13,098).

Stem terms	Class	Proportion, n (%)
**cTAKES default (SNOMED-CT^b^)**		
	N/A^c^	6349 (48.47)
	Body structure	1428 (10.9)
	Over two categories	1265 (9.66)
	Qualifier value	935 (7.14)
	Clinical finding	878 (6.7)
	SNOMED-CT model component	723 (5.52)
	Procedure	721 (5.5)
	Environment or geographical location	217 (1.66)
	Physical object	206 (1.57)
	Substance	143 (1.09)
	Other	233 (1.78)
**RadLex**		
	N/A	6893 (52.63)
	Clinical finding	1839 (14.04)
	Imaging observation	1508 (11.51)
	Process	1000 (7.63)
	Anatomical entity	997 (7.61)
	Property	295 (2.25)
	RadLex descriptor	248 (1.89)
	Object	210 (1.6)
	Procedure	91 (0.69)
	Imaging modality	11 (0.08)
	Nonanatomical substance	5 (0.04)
	Report component	1 (0.01)
**cTAKES default (SNOMED-CT)+RadLex**		
	cTAKES+RadLex	9411 (71.85)
	N/A	3687 (28.15)

^a^cTAKES: clinical Text Analysis and Knowledge Extraction System.

^b^SNOMED-CT: Systematized Nomenclature of Medicine-Clinical Terms.

^c^N/A: not applicable.

## Discussion

### Overview

In this study, we first constructed RadLex-based NER tools for mining free-text radiology reports and evaluated the coverage of the pipelines (step 1). Second, we built a CtED extracted from the FNs of step 1 to improve performance (step 2). Third, we defined OR and MR to consider the potential of expanding the dictionary using RadLex ontology (step 3).

### Performance Evaluation of Each Pipeline (Step 1 and Step 2)

First, the performance of cTAKES+RadLex+GPD was 30.9% (precision 73.3% and recall 19.6%) on its own and 63.1% (precision 82.8% and recall 51%) with the CtED. The CtED for compound terms increased the F-measure by 32.2%, but the F-measure was not obviously changed by the GPD (31.4% vs 31.5%). This indicated that the GPD did not cover the specific compound terms in radiology reports different from the single words. The merit of using RadLex is that we can use the standard vocabularies and relationships such as *Is-A* and *May_cause*. RadLex provides 15 concepts under the top entity, which can assign labels such as *anatomical entity* and *clinical finding* to each entity.

Our tool using cTAKES was able to customize dictionaries by creating a BSV file, which provides a convenient way to leverage those vocabulary resources that are not covered by the default dictionary. In addition, the BSV file stores IDs that can be used to track the parent concepts for a particular term, which enables the classification or profiling of extracted terms using high-level concept classes defined in a vocabulary.

### Stem Term Analysis for Expanding Dictionary (Step 3)

The OR provides profiles of *demand and supply* for stem terms in the corpus. For example, the stem terms of *disease* (PET), *node* (PET), *effusion* (x-ray), and *tube* (x-ray) had a high OR value in both TPs and FNs (Table 4). This means that creating compound terms with high OR–value stem terms in FNs potentially improves precision for capturing entities in each modality’s reports compared with the effort of applying the other vast vocabularies in the pipeline. In addition, the features of the FNs also showed that 97.78% (41,921/42,871) of the compound terms consisted of 2-4 words. This fact suggests that NER performance can be effectively improved by identifying *1 to 3 modified words* and *stem term from each imaging modality.* With regard to the MR, RadLex improved 20.33% of the connectivity with stem terms in the FNs compared with the cTAKES default (SNOMED-CT). The contribution of the improvement can provide criteria in terms of whether we should add phrases to RadLex or to SNOMED-CT. Therefore, stem term–related information such as OR and MR would contribute to expanding dictionaries that have ontological structures. This kind of dictionary-based NER would provide ontology-based benefits such as reasoning concepts and using standard codes and vocabularies. Although it is known that CRFs achieve a higher F-measure than dictionary-based approaches, CRFs generate entities that have no hierarchical structure and relationships.

In contrast, our approach is based on an ontology, which enables interoperable processing and data mining of reports. For example, when we identify the term *pleural effusion*, RadLex ontology can guide us to the parent class *effusion* so that we can finally reach the *Clinical findings* tracking upper concepts. RadLex can also provide relationships such as *pleural effusion may cause of vascular cut-off sign*.

### Limitations

The limitation of this study is that our pipeline is optimized for identifying short compound terms because we divided compound terms using stop words such as *and*. For example, we set the stop word *and* so that we lead to separate the compound term *abdomen and neck* into *abdomen* and *neck*. This approach has the merit of identifying as possible as the stem term, splitting the long phrase *right pleural effusion and left lung pneumothorax* into *right pleural effusion* and *left lung pneumothorax*. Therefore, in the case of capturing long compound terms, we need to combine short phrases. Generally, noun phrase identification for free-text radiology reports is considered difficult because there are many variants of long compound terms. We believe that our method has the potential to capture long compound terms when applying a combination of single and short compound terms.

### Future Work

The annotation tool GATE that we used can identify a partial match with TPs, which means that the types of NER are the same, but the span is not the same. In this study, such partial positives were treated as FNs. We reviewed these uncertainty negatives based on the rule of the stem words and found that 35.4% (90/254) of the partial positives had the potential to change into TPs. This was equivalent to 0.7% of the increased F-measure (cTAKES+RadLex+GPD+CtED). The details of the partial match require further analysis.

The study by Jiang et al [27] demonstrated a state-of-the-art text-mining tool of the Stanford Parser. The study’s results showed that POS-based grammatical approaches are efficient in capturing named entities in free-text radiology reports. In future work, we will extract the POS information to define a pattern of the modified words of the compound term.

Lately, Word2Vec technology has been explored for generating synonyms and expanding the radiology-specific dictionary [28,29]. These studies claimed that a machine learning technology such as Word2Vec supports the building of enhanced dictionaries and reduces the annotation cost. We agree with this claim and believe that it is useful to use Word2Vec to calculate vectors of single terms in the noun phrase, creating modifiers for each stem term. In future work, we will generate modified words using this type of machine learning approach. The customized text-mining tool combined with machine learning technology can help further extract features from radiology reports.

### Conclusions

In this study, we developed a customized NER tool based on RadLex for the recognition of technical terms. We demonstrated that the CtED and stem term analysis have the potential to improve the performance of the dictionary-based NER with regard to expanding vocabularies.

## Abbreviations

BSV: bar-separated value
CRF: conditional random field
CT: computed tomography
cTAKES: clinical Text Analysis and Knowledge Extraction System
CtED: compound terms–enhanced dictionary
EMR: electronic medical record
FN: false negative
FP: false positive
GATE: General Architecture for Text Engineering
GPD: general purpose dictionary
MR: matching ratio
MRI: magnetic resonance imaging
NER: named entity recognition
NLP: natural language processing
OR: occurrence ratio
PET: positron emission computed tomography
POS: parts of speech
SNOMED-CT: Systematized Nomenclature of Medicine-Clinical Terms
TP: true positive
UMLS: Unified Medical Language System
